# Case Clozed: Young Children Can Explicitly Predict Upcoming Words in a Naturalistic, Story-based Cloze Task

**DOI:** 10.1017/S0305000925100251

**Published:** 2025-10-01

**Authors:** Briony Waite, Anthony Yacovone, Jesse Snedeker

**Affiliations:** 1 Department of Psychology, Harvard University, Cambridge, MA, USA; 2 Department of Linguistics, Boston University, Boston, MA, USA

**Keywords:** prediction, discourse, developmental psycholinguistics

## Abstract

Prediction is a central feature of mature language comprehension, but little is known about how and when it develops. This study investigates whether lexical prediction emerges before seven using a novel, naturalistic cloze task. Five and six-year-old children listened to a storybook and occasionally guessed which word might come next. We selected 180 words from the story that were shown to be more or less predictable in a prior cloze norming task with adults. We found that children frequently guessed the correct word or provided an alternative that was semantically related to the target, demonstrating an ability to use the context to explicitly predict upcoming words. Six-year-olds were more accurate than 5-year-olds. These findings show prediction is present (but still improving) in early childhood, motivating future work on the role of prediction in children’s comprehension and learning. Finally, we demonstrate that it is feasible to collect cloze values from children.

## 1. Introduction

Decades of psycholinguistic research have shown that prediction is a central feature of mature language comprehension. Prediction can be understood as the process of activating the meaning (and sometimes the form) of an upcoming word *before* it appears in the linguistic input (see Kuperberg & Jaeger, 2016, Section 3, p.39). When a word can be accurately predicted, it is easier to access and make sense of in that context. For example, in reading studies, adults read predictable words more quickly and are more likely to skip over them relative to less predictable words (e.g., Ferreira & Henderson, 1990; Frisson et al., 2017; Holmes et al., 1989; for a review, see Staub, 2015). In electroencephalography (EEG) studies, the amplitude of the N400 – an event-related potential (ERP) component which indexes ease of lexico-semantic access – becomes smaller as word predictability increases (e.g., DeLong et al., 2005; Kutas & Hillyard, 1980, 1984; Payne et al., 2015; Van Petten, 1993). Prediction research initially focused on comprehenders’ expectations about the kinds of meanings that are likely to be encountered in the upcoming input; however, EEG evidence has shown that linguistic predictions can occur with a greater degree of specificity, resulting in the pre-activation of particular lexical items and their word forms (DeLong et al., 2005; Ito et al., 2016; Kim & Lai, 2012; Laszlo & Federmeier, 2009; Li et al., 2022; Wang et al., 2024; Wicha et al., 2004; Yacovone et al., 2025). So, how and when does prediction at this level of specificity develop?

The present study explores this question by assessing the degree to which five and six-year-old children can predict and subsequently produce words that are likely to appear next in a children’s story. Prior developmental work using the visual-world paradigm has shown that young children can use the meaning of an unfolding sentence to anticipate participants in an event that have yet to be mentioned – for example, shifting their gaze to edible things after hearing the verb *eat* (e.g., Borovsky et al., 2014; Mani & Huettig, 2012; Nation et al., 2003). While these data patterns are often interpreted as evidence of linguistic prediction (e.g., Reuter et al., 2019; Sommerfeld et al., 2023), such findings are ambiguous with respect to *which* representations are being predicted. Is it the lexical item (word), the concept, the referent, or a set of semantic features?

Only a handful of studies with children have used manipulations that unambiguously assess *lexical* prediction (i.e., the prediction of specific words). These studies either conclude that young children fail to predict upcoming words (e.g., Gambi et al., 2018) or present data patterns in which the critical effect occurs late enough to be plausibly attributed to initial bottom-up lexical activation followed by the rapid integration of the preceding context (e.g., Lew-Williams, 2017; Lew-Williams & Fernald, 2007). Critically, prior studies of prediction in children have used stripped-down contexts in which the set of possible referents is limited to the two to four objects that are visually present. The current study explores whether five and six-year-old children can predict words in a natural story using an explicit continuation task that allows the child to choose any word from their lexicon. While this task is more open-ended and complex than those used in prior studies, it may better reflect the complex contexts in which everyday prediction occurs.

Given the ubiquity of prediction in adult language comprehension, it may seem like a foregone conclusion that young children also engage in top-down lexical prediction. There are at least three reasons, however, to expect that lexical prediction might be limited or absent in children under seven. First, some theorists have proposed that becoming a skilled reader results in a reorganization of the phonological representations that facilitates the prediction of lexical form (Mani & Huettig, 2014). Because five and six-year-old children have much weaker reading skills than adults, we would expect, on this hypothesis, to see weaker prediction as well. Second, lexical prediction arises when high-level representations of meaning and context are used to constrain lower level lexical expectations. Thus, prediction is likely to depend on the white matter tracts that connect language areas in the frontal lobe to language areas in the temporal lobe (e.g., the arcuate fasciculus). Myelination of these tracts begins in infancy but continues into adolescence (Skeide et al., 2016), raising the possibility that robust lexical prediction might be delayed until these pathways are sufficiently mature. Finally, prior work with second language learners suggests that lexical prediction may only emerge with high levels of language experience and mastery. Even adults with considerable experience in a second language do not pre-activate the form of highly predictable words (Ito et al., 2017, 2018; Ito & Pickering, 2021). Some studies of second language comprehension find effects that could reflect semantic prediction, but these effects are typically delayed or reduced when compared with first language comprehension. One possible explanation of these findings is that lexical prediction only emerges after one has a considerable amount of language experience (e.g., 10 years or more of immersion). If this were the case, we would expect to see limited lexical prediction in young children. Alternatively, these findings could reflect the primacy of early form to meaning mappings.

In the remainder of this Introduction: (1) we review the prior literature on prediction in young children that uses the visual-world or looking-while-listening paradigms; (2) we briefly discuss sentence completion tasks, noting how the methods and stimuli from this body of work impose limitations on the ages that can be feasibly tested and the conclusions that can be drawn; and (3) we discuss the goals of the present study.

### 1.1. Prediction of meaning and form in child comprehenders

Prediction in adults has been assessed using various measures, including anticipatory looking in visual world studies, early fixations during reading, reaction times, and modulations of ERP components like the N400 (for reviews, see Ito, 2024; Kuperberg & Jaeger, 2016; Ryskin & Nieuwland, 2023; Staub, 2015). In contrast, research on prediction in children relies primarily on anticipatory looks to referents that can be inferred based on the sentence and the visual context (e.g.,; Borovsky et al., 2014; Kidd et al., 2011; Lukyanenko & Fisher, 2016; Mani & Huettig, 2012, 2014; Nation et al., 2003; Özge et al., 2019; Reuter et al., 2019; Sommerfeld et al., 2023).

In a typical visual-world anticipatory looking study, a child hears a sentence containing information that could potentially allow them to predict an upcoming noun, and researchers measure their looks to a picture or object that is a potential referent of the target noun before the noun is spoken aloud (Borovsky et al., 2012, *inter alia*). For example, Borovsky and colleagues (2012) explored the role of subject and verb constraints on anticipatory looking in children between the ages of 3–10. Participants saw a display with four images while listening to a sentence describing an event (e.g., “The pirate hid the treasure”). The target image depicted the direct object of the sentence, which was the theme of the event (the treasure). This word was predictable based on the combination of the agent (the pirate) and the action (hid). The other three images were possible themes that were related either to the agent (a ship), the action (a bone that might be hidden by a dog), or neither (a cat). Both the adults and the children showed anticipatory looks to the target (the treasure) after hearing the agent and the action (“The pirate hid…”), suggesting that they were able to use the linguistic context to anticipate a likely upcoming theme. Interestingly, this effect varied not with age but with relative language ability (as compared to age-matched peers).

There is, however, an inherent ambiguity in findings of this kind. While these anticipatory looks could reflect prediction of the lexical label, they could also reflect prediction of the concept, the referent itself, or even an inference about the event under discussion (with no expectation that the object in question will be explicitly mentioned). For example, a young child who hears a sentence like “What did the pirate hide?” will likely look to the treasure after hearing the verb, even though the actual word (*treasure*) would be unlikely to occur in this syntactic context (Atkinson et al., 2018; Seidl et al., 2003). This ability to infer missing properties of an event is not unrelated to prediction – without those event inferences, there would be no basis for predicting the upcoming referent, concept, or lexical item. It is possible, however, to make event inferences without predicting the word itself. Thus, while studies of this kind provide valuable insights into children’s inferential processes, they do not provide definitive evidence of prediction at the lexical level.

To the best of our knowledge, there are two kinds of looking time studies with children that could potentially demonstrate lexical prediction: The first kind explores whether children who hear gender marked, pre-nominal articles are faster to look to the target referent when the competitor’s label was of a different, mismatching grammatical gender (Lew-Williams, 2017; Lew-Williams & Fernald, 2007). These studies find that, at 3, 6, and 10 years of age, native speakers make rapid use of gender marking to correctly identify the target referent. In children, however, the critical gaze patterns emerge *after* the onset of the target noun, making them consistent with either a bottom-up integrative process or a top-down predictive process. The second kind of relevant looking time study explores whether children (aged 2–5 years) use the phonological form of a pre-nominal, indefinite determiner to predict an upcoming noun, for example, “Can you see *an*…ice cream cone?” (relative to *a*…ball). Specifically, Gambi and colleagues (2018) had participants look at a screen with two images while listening to their critical sentences. In a semantic control condition, participants saw a pair of objects on one side (two ice cream cones) and a singleton object on the other (one ball). Then, they heard a sentence instructing them to look at one of the images (“Can you see *one* …ball?”). Children as young as two began looking to the correct side before the onset of the noun, indicating that the pre-nominal quantifier could be used to identify the reference set (see also Huang & Snedeker, 2009). In the critical phonological condition, there were two singleton objects on the display: One with a label beginning with a vowel (an ice cream cone), the other with a label beginning with a consonant (a ball). Then, the participant would hear an instruction with a pre-nominal determiner that was compatible with only one of the words (“Can you see *a* …ball” or “Can you see an…ice cream cone?”). A pause was inserted after the determiner, giving participants over a second in which they could generate expectations about the upcoming noun. Adults were able to use the determiner to predict the correct referent and shift their gaze appropriately, but children (aged 2–5 years) could not.^1^ The authors concluded that the ability to generate narrow, lexically-specific predictions develops later than semantic-based prediction.

These kinds of visual-world studies have one further limitation – the set of possible referents varies from two to four objects. Thus, *prediction* in these studies is more akin to a *selection* process (i.e., using the available cues to pick the most likely or relevant object from a small set of options). This limitation is critical for two reasons: First, selection of this kind can be carried out by mechanisms that might not be predictive. For example, comprehenders might evaluate a determiner against the object that they are currently fixating and then shift their gaze elsewhere if the pair is contradictory. Second, if prediction only occurs in these highly constrained visual-world experiments, it would suggest that predictive processes are of little use to children in their daily lives, as naturalistic contexts typically allow for a wider range of possible (but perhaps improbable) outcomes. In sum, while there is ample evidence for anticipatory looking in children, there is no clear evidence for lexical prediction per se. There is, however, another literature that is rarely discussed in the context of predictive processing, which at first glance appears to be relevant for these questions – namely, the work that uses sentence completion or elicitation tasks to assess children’s syntactic knowledge and reading ability. We will review this work next.

### 1.2. Elicitation tasks as a measure of lexically specific linguistic prediction

Psycholinguists typically assess lexical predictability using a cloze production task (see Taylor, 1953). The procedure is simple: each participant receives written sentences or passages with one or more words missing, and they are asked to provide the missing words. Cloze values are then calculated for each target word. If 80 out of 100 people provided the correct word, then that word (in that context) has a cloze probability of 80%. If only 5 out of 100 participants provided the correct word, then that word would be considered less predictable with a cloze value of 5%. Most contemporary psycholinguistic studies only provide the context prior to the missing target word, simulating the amount of information available to the comprehender before they encounter the word in the input. These cloze values strongly correlate with neural responses associated with lexico-semantic access (the N400) and reading times in adults and thus have long been used as a metric of the predictability of a word given its preceding context (Brothers & Kuperberg, 2021; Kutas & Hillyard, 1984; Staub, 2015). Cloze values are typically collected using written materials, making them difficult to implement with young children. Thus, developmental researchers generally rely on cloze values from adults when constructing stimuli for children. To the best of our knowledge, only one study has conducted a cloze task with children for psycholinguistic research (see Lee et al., 2023).

There are, however, two separate literatures that use similar paradigms with children, albeit for very different purposes: First, fill-in-the-blank tasks are often included in standardized measures of reading comprehension. The task in these assessments is very similar to a traditional cloze task – children are given sentences or passages with certain words removed and are tasked with filling in the missing words (e.g., Oakhill, 1997; Woodcock, 2011). Some tests use a multiple-choice format, while others allow test takers to fill in a blank with any word they choose. For example, the Woodcock Reading Mastery Test Revised (WRMT-R) includes a comprehension task in which children read a passage or sentence with a missing word and fill in the blank with any word they choose, much like a standard cloze task. To the best of our knowledge, these kinds of elicitation tasks have not been used to explore spoken language comprehension. In contrast with our study, they use passages constructed for the test rather than ecologically valid stimuli. These tasks also do not seek to compare predictions in young children and adults. Nevertheless, the successful implementation of these tasks in widely used assessments demonstrates that cloze procedures can be used with children who are old enough to read.

The second literature comes from the field of language acquisition, where sentence completion tasks are used to measure children’s morpho-syntactic knowledge. For example, Berko (1958) asked 4- and 5-year-old children to complete spoken utterances like “This is a wug. Now there is another one. There are two of them. There are two ____.” While the spoken nature of this task is similar to the current study, these paradigms, by design, involve formulaic templates and repetition of the relevant base word, and are thus not directly relevant to our research question.

To the best of our knowledge, only one study has tested children in a cloze task with the goal of understanding children’s predictive processing. Lee and colleagues (2023) used a speeded sentence completion task with 4- to 12-year-old children (*Mean Age* = 9) to investigate whether their predictive processing exhibits the reaction time signatures that characterize a race process (i.e., where multiple lexical candidates are considered for a given context, gaining activation until one reaches the threshold for production). Race models have been argued to account for lexical selection (and the predictive processes that precede selection) in adults (see Staub, 2015). Lee and colleagues (2023) tested children’s ability to quickly complete sentences that originally had either more or less predictable endings and then compared their performance to that of adults. Critically, in this study, all participants were literate and read sentence fragments word-by-word and then produced a verbal completion as quickly as possible. Lee et al. found a developmental difference in reaction times (i.e., adults were overall faster than children) that may indicate improvement in generating predictions. But this difference could also have been due to improvements in text decoding or task switching (from reading to production). Also, while the sample included children as young as four, the mean age was nine, and no further age breakdown was provided. The authors also do not report overall accuracy (i.e., the ability to correctly guess the original ending from the selected stimuli) nor accuracy differences between adults and children. Thus, these results leave open many questions about lexical prediction in pre-literate children and how predictive processing in children compares to that in adults.

### 1.3. The present study

The present study uses a spoken cloze task to assess lexical prediction in 5 and 6-year-old children. Specifically, we use naturalistic materials to explore prediction in the context of a single extended discourse (a cartoon story) rather than in isolated, carefully constructed sentences like those used in typical psycholinguistic studies (e.g., cloze tasks or visual-world studies). With these materials, we can study lexical prediction in a context that closely resembles one that children encounter in their daily lives. Critically, lexical prediction in this story-based context is not limited to a small set of candidate words or referents (as in the visual world paradigm). Rather, children are free to use any word from their lexicon. We ask three main questions using this paradigm: How does performance change with age? What features of a word make it easier or harder to guess? And when children do not guess the exact word, do their responses indicate sensitivity to the context? To preview our results, we find evidence of robust lexical prediction in both adults and children, as well as preliminary evidence for developmental improvement between the ages of 5 and 6.

## 2. Method

### 2.1. Participants

Our final sample included 45 adults (*Mean Age =* 23 yrs, *Range* = 16–40 yrs, 11 male, 31 female, 3 not reported) and 45 children (*Mean Age* = 6;0 yrs (yrs;mos), *Range* = 5;0–6;11, 26 male, 19 female) from the Harvard University community and the greater Boston area. We chose to preregister a relatively large sample size (in the context of developmental research) because we had no prior data with which to estimate effect sizes. All participants were native English speakers who had been exposed to English since birth. Adult participants provided written consent, and those under the age of 18 verbally assented and received written parental consent to participate. We compensated adult participants with course credits and child participants with gift cards. Three additional participants (one adult and two children) were tested but excluded following our pre-registered criteria (https://osf.io/dgbv3/).

### 2.2. Procedure

Participants played an online game with an experimenter over Zoom. This game was a spoken version of the classic cloze task (Taylor, 1953) that we adapted for use with children. Participants watched a cartoon narration of a children’s book and occasionally guessed the next word in the story.

**Figure 1.**
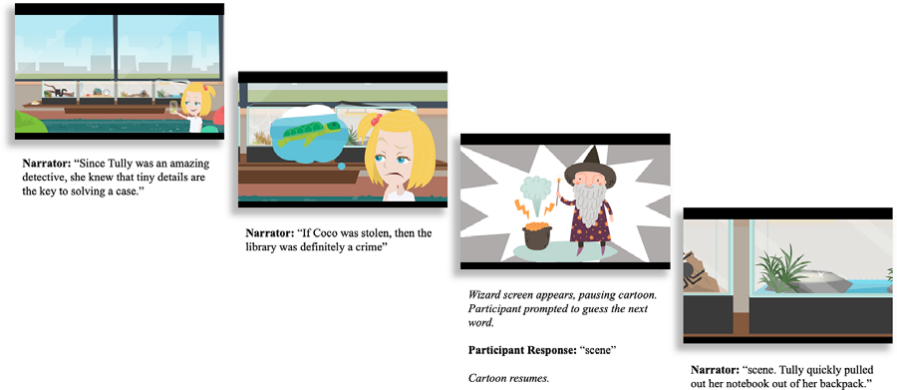
An example of a single trial in the story-based cloze task. Pictures show the visual information presented to participants, and text shows corresponding transcriptions of the spoken context. The first two panels show still frames from the cartoon before a target word. The third panel shows the guessing frame and participant response. The final panel shows the cartoon continuation, starting with the target word.

At the start of the testing session, the experimenter met participants on Zoom and helped them set up their computers so that they could see and hear the cartoon. The cartoon was presented via PsychoPy (Peirce et al., 2019) on the experimenter’s computer and screenshared with participants. Participants were told that they would be watching a 30-minute cartoon and that occasionally a wizard would appear on the screen and “freeze” the video. To “unfreeze” the video and continue watching, they had to tell the wizard which word they thought came next in the story. Participants were instructed to say a single word rather than completing the rest of the sentence and were told that there were no right or wrong guesses; the cartoon would resume even if the actual story continuation differed from their guess. After the participant provided their guess, the wizard disappeared and the cartoon resumed, revealing the correct word in the story continuation (see example procedure in Figure 1).

The cartoon always paused immediately before the onset of the target word. If necessary, we trimmed the audio file to remove any co-articulatory information or initial voicing of the target word’s onset. In addition, when the cartoon paused, the still image of the cartoon was replaced with an image of the wizard.

Before beginning the study, participants watched a 2-minute cartoon of the *Three Little Pigs* and practiced guessing eight words. Some of these target words were highly predictable, and thus easy to guess (e.g., “The big bad *wolf*”). Other target words appeared in less constraining contexts, meaning that there were several possible continuations (e.g., “The wolf climbed down and hurt his *tail*”). These practice trials also demonstrated to participants that the cartoon would resume even if their guess differed from the story continuation. Each participant guessed 60 words during the critical story and answered six comprehension questions at the end.

### 2.3. Stimuli

We were interested in how well adults and children predicted a set of 180 target words from our selected story. Participants did not guess all 180 target words; rather, we created three versions of the experiment with 60 target words each. All target words were common nouns that we selected based on their cloze probability from an earlier cloze task in which adult participants read the story in its entirety and guessed a subset of words (see below). Based on these values, we selected 90 target words with higher cloze values (*M* = 81.2%, *Range* = 53–100%) and 90 target words with lower cloze values (*M* = 7.2%, *Range* = 0–50%). We next provide more information about the story, cartoon, and target words.

*Selecting our story and creating the cartoon stimulus.* Participants listened to an abridged version of the children’s book *Mystery of the Turtle Snatcher* by Kyla Steinkraus and watched an accompanying cartoon created by the second author (AY). We selected this book for two reasons: First, the story is unlikely to be familiar to participants. None of our participants reported having heard it previously, ensuring that correct guesses reflect an understanding of the unfolding discourse rather than prior exposure. Second, the story uses age-appropriate language and discusses topics familiar to young children. This story is rated at a third-grade independent reading level. We selected a story with a reading level slightly above the age of our child participants because such a gap is typical of material read aloud by teachers and parents to young children. Stories that are intended to be read by 5- and 6-year-olds focus on the decoding of text, and thus are short and highly repetitive, making them unsuitable for our research questions. As we will demonstrate, our participants clearly comprehended the story.

We shortened the original text to present the entire story within a single testing session. The story was recorded by the second author (AY) at a natural speaking rate of roughly 3.25 words per second. He also created a cartoon to accompany the story using Vyond animation software (Vyond.com). In this cartoon, we aimed to illustrate the action of the story as it progressed without revealing events before they occurred in the narrative. The final set of target words was not known at the time the cartoon and recording were made; thus, we did not knowingly emphasize or privilege those words in ways that would artificially enhance their predictability.

*Determining the predictability of all words in the story.* Before selecting our 180 target words, we first conducted a written cloze task with adults on Amazon Mechanical Turk to determine the predictability of every word in the story. Participants read portions of the story while either guessing words or simply reading for comprehension. During the guessing portions, participants guessed a single word, saw the correct word, and then guessed the next word. This word-by-word guessing continued for approximately 300 words, after which they continued reading sentence-by-sentence. Participants guessed one of 15 sections and read the remaining 15. We collected data from 541 participants and excluded 91 for failing data quality checks, resulting in a final sample of 450 participants and 30 observations per word.

To find written cloze values for each word in the story, we calculated the proportion of participants who provided the correct response for each word. For example, if six out of 30 participants provided the correct guess for a particular word, that word would have a cloze value of 20% in that context (6/30). Note that we were interested in the proportion of participants who provided the *correct* word, rather than the word provided by the majority of participants. This approach could, therefore, result in low cloze words (words that few, if any, participants guessed) with a high cloze competitor (a word that many, or all, participants provided). We return to this issue in Results (see Section 3.1).

*Selecting target words.* All 180 target words were common nouns (e.g., *turtle*, *friends*, *book*). There was never more than one target word in a single sentence. We did not limit our targets to sentence-final words, so they appeared in various sentence positions. Because we are using a long, cumulative discourse, there were some target words that appeared many times in the story. However, we never chose a single word as a target more than three times, and these repetitions were split across different versions of the experiment, ensuring that no participant was asked to guess the same target word more than once.

### 2.4. Data coding and analyses

We recorded participants’ guesses during the online session and coded them offline. Guesses were coded as correct if they exactly matched the target word (e.g., target = *turtle*, response = *turtle*) or if the target word and the guess shared the same lemma (e.g., target = *friend*, response = *friends*) or if the words were compounds with the same first morpheme (e.g., target = *bookshelves*, response = *bookcases*). If a participant guessed more than one word, we only coded the first content word. However, if the participant simply repeated the last word from the story in their response, for example, after hearing “Tully handed the invitations to her four best…” the participant responded “best friends,” we coded the new word (*friends*) as their guess. If a participant gave an unintelligible response, the experimenter prompted them to repeat it. If the coder could not understand the response from the recording, that item was excluded from our analyses.

We also coded the syntactic category of the guess and its semantic relationship to the target word. We were interested in three broad categories of semantic relationship: exact match, semantically related (synonyms, hypernyms, hyponyms, and co-hyponyms), and unrelated (generics, other). Examples of responses in each category are shown in Table 1.

**Table 1.** Examples of match, semantically related, and unrelated responses

Category label	Sentence from the story	Target word	Response
Exact match	Tully handed the invitations to her four best…	Friends	Friends
Semantically related categories			
Synonym	She climbed over her clothes, slid down the other side to the…	Floor	Ground
Co-hyponym	He glanced at Tully and then looked down at his…	Hands	Feet
Hypernym	They are only found in a certain…	River	Place
Hyponym	I was hoping you might be able to fill in for me today, maybe after…	School	Class
Unrelated categories			
Generic	Tully fixed his lid, then got the plastic container full of…	Crickets	Stuff
Other	Emily snapped the…	Book	Teeth

### 2.5. Data exclusion criteria

We excluded two children for answering more than three of the six comprehension questions incorrectly. Overall accuracy from children in the final analysis was 80%. No adults were excluded on the basis of comprehension accuracy, as their overall accuracy was 98%. We also planned to exclude participants who gave the same, contextually irrelevant response on more than three consecutive trials, but no one used this strategy. We excluded one participant who experienced technical difficulties.

## 3. Results

We address four questions in our analyses: First, how does performance on the cloze task vary by age group (adults and children) and by our a priori categorization of predictability (high cloze and low cloze)? Second, do any lexical features of our target words (e.g., frequency, concreteness, age of acquisition) explain participants’ accuracy in predicting them? Third, when participants guess incorrectly, are their responses meaningfully related to the correct target word? Finally, how do visual information and word repetition influence accuracy? The questions addressed in Sections 3.1, 3.2, and 3.3 were all pre-registered (see https://osf.io/dgbv3/ for details), and all other analyses (the sub-analyses in 3.1, and those in 3.4) should be treated as exploratory. Analyses were conducted in R (R Core Team, 2024) using the *lme4* package (Bates et al., 2015). Further details of each analysis are presented in the sections below.

### 3.1. How does cloze performance vary across age and normed word-level predictability?

**Figure 2.**
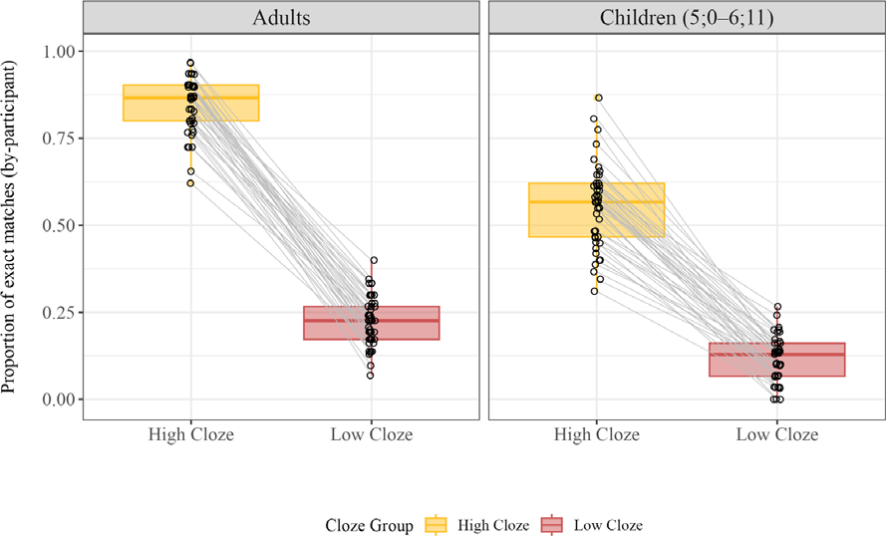
Proportion of exact matches across Age Group and Cloze Group. Each dot represents a single participant. Lines between dots link participant data for high and low cloze words.

We first investigated the overall performance of adults and children in our spoken cloze task. As shown in Figure 2, both adults and children often made accurate lexical predictions. Adult participants correctly guessed 85% of the high cloze target words and 22% of the low cloze words. While children were less accurate, their performance was nonetheless impressive: They correctly guessed 56% and 12% of the high and low cloze words respectively.

To explore the significance of these patterns, we implemented a generalized logistic mixed effects model regressing Accuracy (binary score, 1 = match, 0 = no match) onto fixed effects of Age Group (*child* = −0.5, *adult* = 0.5) and Cloze Group (*high cloze* = 0.5, *low cloze* = −0.5), as well as their interaction. The model had a random intercept and random slope of Cloze Group for participants, and a random intercept and random slope of Age Group for items. There were main effects of Age Group (*b* = 1.6, *SE* = 0.17, *z* = 9.49, *p* < .001) and Cloze Group (*b* = 4.38, *SE* = 0.33, *z* = 13.16, *p* < .001), as well as a significant interaction between them (*b* = 1.46, *SE* = 0.36, *z* = 4.02, *p* < .001).

We conducted planned pairwise comparisons using the *emmeans* package (Lenth, 2024) between the two age groups (within each cloze group) to unpack this interaction. Results indicated significant differences between adults’ and children’s accuracy in guessing high cloze words (*b =* 2.34, *SE* = 0.23, *z* = 10.17, Tukey-adjusted *p* < .001) and low cloze words (*b* = 0.87, *SE* = 0.27, *z* = 3.28, Tukey-adjusted *p* = .001). The difference between adults’ and children’s accuracy in guessing low cloze words, however, was less than the difference for high cloze words, resulting in the observed interaction from the main model. This pattern is what we would expect under the following conditions: prediction improves across development, adults are better at predicting than children, and the low cloze words are simply less predictable for everyone (and thus provide fewer opportunities to demonstrate differences in prediction).

*Quantifying performance level.* The analyses above do not provide a satisfying answer to the question of *how* good (or poor) children are at making predictions. One tool that psychologists use to measure task performance is d-prime values, which are calculated by comparing how often a person gives a particular response when it is correct (a hit) relative to how often they give that response when it is incorrect (a false positive). D-prime has long been used as a measure of sensitivity in studies of perception and decision making (see Macmillan, 1993).

**Figure 3.**
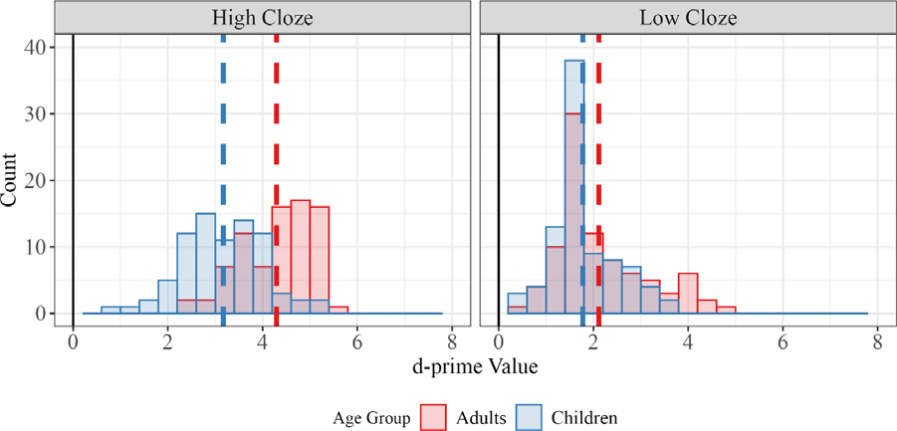
D-prime values for each target word in the story. A d-prime value of 0 indicates chance (black lines). Mean d-prime values for high and low cloze targets are shown by the dash lines in red (adults) and blue (children).

We calculated d-prime for each target in each population (Figure 3). For example, children’s d-prime for the target word *dog* is the difference between their hit rate (correctly guessing *dog*) and false alarm rate (guessing *dog* when the target word is not *dog*). Higher d-prime values indicate greater sensitivity to the signal, whereas a value of zero indicates random guessing. Mean d-prime values for children in our task were 3.16 (*SD* = 0.86) for high cloze and 1.78 (*SD* = 0.71) for low cloze. Mean adult d-prime values were 4.3 (*SD* = 0.74) for high cloze and 2.12 (*SD* = 0.97) for low cloze. Thus, adults and children showed substantial sensitivity to the contexts in which individual words were used, indicating that their overall performance was quite strong.

*Cloze versus constraint.* The prior analysis focused on how accurately participants were at guessing the exact target word from the story. While this approach reveals how often participants guessed the word that the author chose, it may conceal instances of strong prediction where participants converged on a different word. The present analysis, therefore, explores how often participants within a group converge on the same response, regardless of whether that response is correct. To do so, we explore contextual constraint. The constraint of a context can be understood as the degree to which participants converge on a single word, or the cloze probability of the most commonly produced (modal) response. High cloze words, by definition, occur in high constraint contexts. But low cloze words can appear in both high and low constraint contexts. If a word is unpredictable in a context that does not generate strong expectations for any single word, it is a low cloze word in a low constraint context. In contrast, if a word is unpredictable in a context that generates a strong expectation for a specific alternative, it is a low cloze word in a high constraint context. These latter words can arise when an author selects a particular word, but readers anticipate another.

For example, consider the following sentences: “For breakfast, I ate… *waffles.*” and “For her birthday, we baked a… *pie.*” If one participant out of ten provided the intended target in each example sentence (*waffles* and *pie*), then both words would have a cloze probability of 10%. The response pattern for the remaining participants, however, might be very different in each sentence. If participants in the first sentence gave many different responses (e.g., *pancakes*, *cereal*, *toast*), then *waffles* would be a low cloze continuation in a low constraint sentence. This is in contrast with the second sentence, where the other participants might all say “cake.” In that case, *pie* would be a low cloze continuation in a high constraint sentence. Participants are not predicting the word that the author intended; however, the high agreement indicates that participants still generated a strong prediction given the preceding context.

To explore the degree to which our contexts were constrained for both adults and children, we calculated the probability of the most common response for each item, independently in each age group. We removed items with no single modal response, that is, there were two or more responses with the highest number of observations. On average, in the high cloze condition, the context constraint values closely mirrored the proportion of correct responses for adults (high cloze accuracy = 85%, high cloze constraint = 84%) and for children (high cloze accuracy = 55%, high cloze constraint = 59%). This is because the most common response was the correct response 94% of the time for adults and 80% of the time for children. In the low cloze condition, the context constraint values were higher on average than the proportion of correct responses for adults (low cloze accuracy = 23%, low cloze constraint = 53%) and for children (low cloze accuracy = 12%, low cloze constraint = 35%). Unlike in the high cloze condition, the most common response was often not the word that actually appeared in the text (only 23% and 17% of the time for adults and children respectively).

To explore whether children and adults converged on the same words, we focused on the subset of cases where there was a continuation that was clearly preferred (chosen by more than a third of the participants in both groups). Thus, we had 64/90 high cloze trials and 17/90 low cloze trials for this analysis. We calculated the percentage of these contexts in which the most common response was the same for both children and adults. For the high cloze items, children and adults largely produced the same response (95%), which was always the correct target word. For the low cloze items, children and adults converged on the same response 65% of the time, and this response was the target in 43% of the cases.

**Figure 4.**
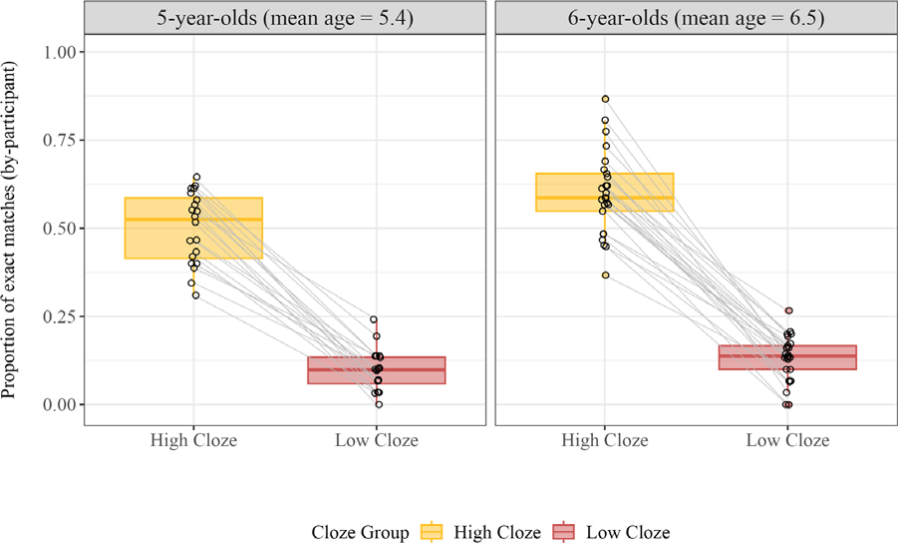
Proportion of exact matches across Age Subgroup and Cloze Group. Each dot represents a single participant. Lines between dots link participant data for high and low cloze words.

*Is there improvement within our child age group?* Children in our study were less accurate than adults, suggesting that the ability to make correct lexical predictions improves across childhood. To explore development on a finer time scale, we conducted an exploratory analysis, splitting our child age group into older (6;0–6;11, *Mean Age* = 6;6) and younger (5;0–5;11, *Mean Age* = 5;5) children. Older children (N = 25) were indeed more accurate than younger children (N = 20), even within our limited age range (see Figure 4). We conducted a generalized logistic mixed effects model with fixed effects of Age Subgroup (*older* = 0.5, *younger* = −0.5) and Cloze Group (*high cloze* = 0.5, *low cloze* = −0.5), as well as their interaction. We included random intercepts for participants and items and random slopes of Age Group and Cloze Group for items and participants respectively. There were reliable effects of Age Subgroup (*b* = 0.67, *SE* = 0.20, *z* = 3.28, *p* < .001) and Cloze Group (*b* = 3.62, *SE* = 0.35, *z* = 10.21, *p* < .001); however, there was no significant interaction between them (*b* = −0.11, *SE* = 0.38, *z* = −0.30, *p* = 0.76). Thus, in this context at least, the accuracy of children’s predictions is improving between the ages of five and six.

### 3.2. Do any lexical features predict accuracy in the cloze task?

Successful prediction in the cloze task requires inferring the meaning based on contextual constraints and then accessing and producing the word form. Thus, we might expect that prediction would depend on some of the same factors that influence word recognition during comprehension and naming times during production. To explore this, we looked at three variables: lexical frequency, Age of Acquisition (AOA), and concreteness. Frequency per million words was obtained from the SUBTLEX_US_ (Brysbaert & New, 2009) corpus of American subtitles and log transformed. We use AOA ratings from Kuperman et al. (2012), who asked adult participants to enter the age at which they believed that they knew the meaning of a given word. Concreteness ratings are from Brysbaert et al. (2014), who obtained participant ratings on a five-point scale from abstract to concrete. Frequency and AoA affect reaction times in both comprehension and production tasks and are believed to primarily influence the difficulty of accessing the form of a word (Graves et al., 2007; Jescheniak & Levelt, 1994; Johnston & Barry, 2006). Concreteness affects memory for words and interacts with other variables in a manner that suggests that it primarily affects access to the concept encoded by the word (Romani et al., 2008).

We constructed a generalized logistic mixed effects model predicting accuracy with fixed effects of Age Group (adults = 0.5, children = −0.5), the *z*-scored lexical predictors (log Frequency, Concreteness, and AOA), as well as the two-way interactions between Age Group and each lexical feature. Random intercepts were included for participants and items, as well as a random slope of Age Group for items. Model results are shown in Table 2.

**Table 2.** Results of the lexical feature model predicting overall accuracy

Effects	Estimate	SE	*z*-value	*p*-value
Age group	1.59	0.19	8.57	> 0.001*
Frequency	1.06	0.26	4.09	> 0.001*
Concreteness	0.52	0.24	2.13	0.03*
AOA	−0.19	0.26	−0.75	0.45
Age group: frequency	0.27	0.19	1.41	0.16
Age group: concreteness	0.06	0.18	0.31	0.75
Age group: AOA	0.05	0.18	0.26	0.79

* >0.05.

There was, unsurprisingly, a robust effect of Age Group. There were also main effects of Frequency and Concreteness, such that both adults and children were more accurate at guessing words that were higher in frequency and concreteness. There was no significant effect of AOA; however, this could be due to the relatively low age of acquisition values in our selected set of target words. Recall that all targets were nouns and appeared in a children’s story, thus limiting the range of age of acquisition values. Our findings suggest that access to both the concepts (concreteness) and the lexical form (frequency) affects the accuracy of prediction. The lack of interactions with age suggests that these factors influence adults and children to a similar degree.

We also ran individual models for each word feature to understand the effect of each feature independently. Details and results from these additional models are in the Supplementary Materials included on OSF (https://osf.io/dgbv3/).

### 3.3. When participants guess incorrectly, are their guesses meaningfully related to the intended target word?

Current psycholinguistic models view lexical prediction as the activation of the relevant concept, based on context, followed by the activation of the semantic and lexical form features associated with that concept. On this theory, if prediction is only occurring at higher levels (concepts, semantic features), it might result in the production of semantically related lexical items in our cloze task. To explore this possibility, we investigated participants’ incorrect guesses, focusing on the degree to which they were semantically related to the target word. For example, if the target word is *dress* and the participant guesses “skirt,” it suggests an ability to use the unfolding context to infer the kind of word that might appear next (even when unable to guess the exact word from the story).

**Figure 5.**
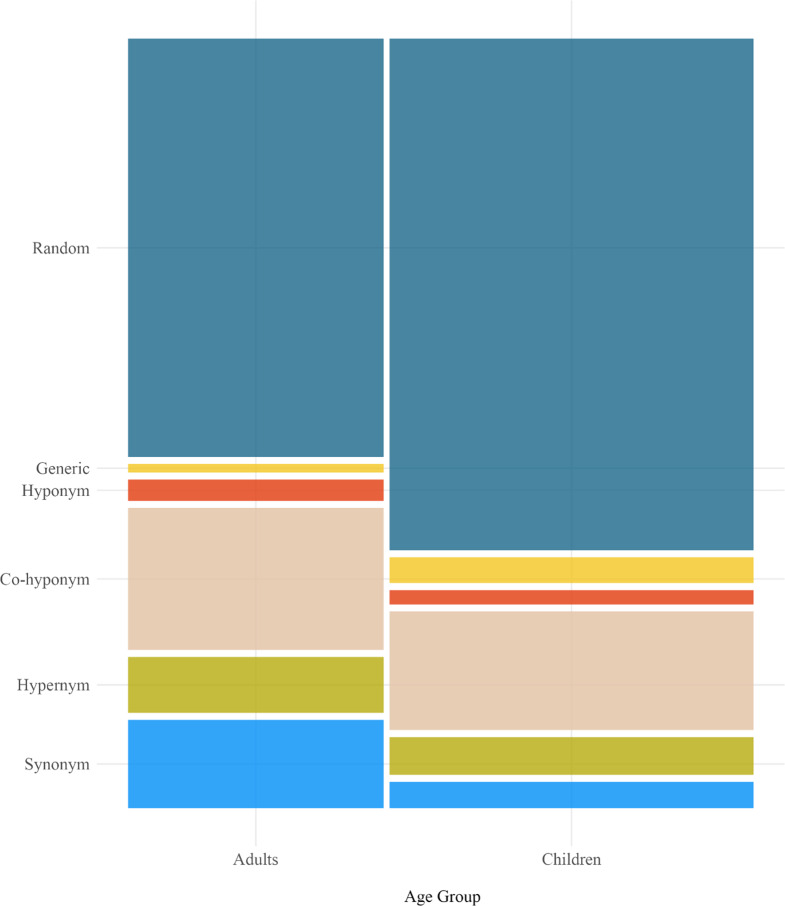
Proportion of non-match response types across age groups. This plot excludes correct responses. The relative size of the panels shows the proportion of responses in each group – the child bar is wider because children provided more non-match responses overall.

To explore semantic relatedness, we looked at each incorrect response and coded its taxonomic relationship, if any, to the target word. Specifically, we coded whether the response word was a synonym, hypernym, hyponym, or co-hyponym of the target word. For example, if the target word was *dog*, then *pup* would be a synonym, *animal* would be a hypernym, *labrador* would be a hyponym, and *cat* would be a co-hyponym. Words that were not semantically related were coded as either a generic response (e.g., *stuff*, *thing*), or random (any response that did not fit into another category). Figure 5 shows the proportion of responses in each category across age and cloze groups.

Our statistical analysis focused on the degree to which incorrect responses were semantically related to the target. To assess this, we removed correct responses from the data sets and coded responses that were taxonomically related as 1 and those that were not as 0. As Figure 5 illustrates that both adults and children often produced a semantically related word when they guessed incorrectly, but adults appeared to do so more often. For high cloze targets, adults and children provided a semantically related alternative 43% and 27% of the time, whereas for low cloze targets, their proportions were lower at 42% and 26% respectively.

To test this pattern, we implemented another generalized logistic mixed effects model with fixed effects of Age Group (adults = 0.5, children = −0.5), Cloze Group (high = 0.5, low = −0.5), and their interaction. We included random intercepts for participants and items, and random slopes of Age Group and Cloze Group for items and participants respectively. Results indicated a main effect of Age Group (*b* = 0.80, *SE* = 0.26, *z* = 3.04, *p* = .002); however, there was no main effect of Cloze Group (*b* = −0.07, *SE* = 0.48, *z* = −0.15, *p* = 0.88), and no interaction between them (*b* = −0.33, *SE* = 0.43, *z* = −0.76 *p* = 0.45). In short, adults were better than children at providing semantically related responses when they were unable to guess the exact word from the story.

### 3.4. Exploring the role of visual context and repetition in cloze performance

The present study used a novel, naturalistic cloze task relying on a cartoon narration of a children’s book. This approach is a radical departure from more traditional cloze tasks using isolated, written sentences or short vignettes. Our approach has both advantages and limitations. We therefore address two points of interest (or concern) in this section: How does the inclusion of a visual stimulus and the presence of an entire preceding discourse influence participants’ predictions? Specifically, did participants find it easier to guess target words when a corresponding object was visible, and did they find it easier to guess target words that had previously been mentioned in the story?

**Figure 6.**
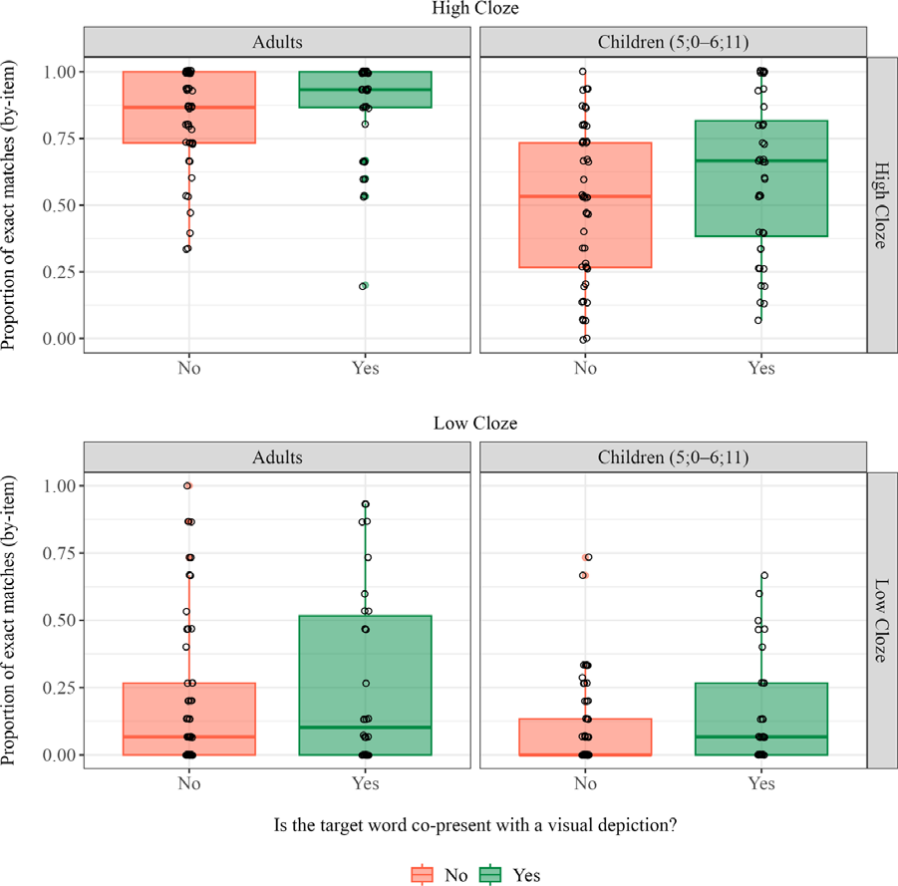
Proportion of correct guesses by visual co-presence. Each dot represents an item. Adult accuracies are shown in the left panel, and child accuracies are on the right. The upper panel shows high cloze items, and the lower panel shows low cloze items.

To address the first question, we conducted an exploratory analysis to check whether seeing a visual depiction of the target word immediately prior to guessing impacted accuracy (Figure 6). We conceptualized co-presence as having a depiction of the target word in the cartoon immediately prior to that word appearing in the input (e.g., an image of a clock on a wall in the background immediately prior to the target word *clock*). We constructed a generalized logistic mixed effects model predicting accuracy with fixed effects of Co-presence (yes = 0.5, no = −0.5), Cloze Group (high = 0.5, low = −0.5), Age Group (adults = 0.5, children = −0.5), and the two-way interactions between Co-presence and Cloze Group or Age Group respectively. We did not include the three-way interaction. Random intercepts were included for participants and items, as well as a random slope of Age Group for items and Cloze Group for participants.

Results indicated a main effect of Co-presence, as both adults and children were slightly more accurate when a depiction of the target was present immediately before they guessed (*b* = 0.75, *SE* = 0.32, *z* = 2.34, *p* = .019). There was, however, no interaction between Age and Co-presence, indicating that adults and children use visual information to a similar degree in this task.

We next asked whether participants were more accurate at guessing words that had been previously mentioned. We considered target words to be mentioned if they had already appeared in the story text before the point at which they were a target. Participants were more accurate in guessing words that had been previously mentioned (adults = 65%, children = 50%) relative to words that were not mentioned (adults = 45%, children = 24%). This difference resulted in a significant main effect of Prior Mention (*b* = −1.80, *SE* = 0.46, *z* = −3.95, *p* < .001), but not an interaction between Prior Mention and Age Group. In other words, children and adults both seem to be using the contextual cue of whether a target word has already been mentioned to a similar degree.

The results of these two analyses demonstrate that both adults and children benefited from the inclusion of the visual cartoon stimulus and the extended discourse context.

## 4. General discussion

In this study, we used an age-appropriate spoken cloze task to investigate whether adults and children (5;0–6;11) can make explicit lexical predictions about the next word in a story. Specifically, we asked three main questions: First, how does performance on this task change with age? Second, which features of a word make it more or less difficult to predict? Third, when children are unable to provide the correct continuation, do their responses still indicate a sensitivity to the unfolding context?

We found that adults were more accurate in their predictions than children. Nevertheless, both groups performed quite well. For the high cloze targets, children produced the correct word, out of the many thousands of words that they know, on roughly half of the trials. Thus, while performance was lower for children than adults, both groups showed strong sensitivity to the differing contexts in which these words occurred (as illustrated by the d-prime analysis). In an exploratory analysis, we found evidence that prediction accuracy improved with age in our child sample: Our 6-year-olds were more accurate than our 5-year-olds. Both adults and children were more accurate at guessing target words that were higher in frequency and concreteness, and when adults and children guessed incorrectly, they demonstrated a sensitivity to the context by often providing words that were semantically related to the target word. When children and adults converged on words other than the target, they often converged on the same word, suggesting that they had a similar understanding of the story (and its contextual constraints).

Our results reveal developmental differences in lexical prediction between 5- and 6-year-olds and from childhood to adulthood. What might account for these improvements? We see at least four possibilities: First, children may be developing better discourse understanding, leading to more accurate expectations about what could happen next in the story. Second, this change may be, in part, due to better evaluation and filtering of one’s own hypotheses about the missing word, perhaps as a result of improving executive function, memory, or metamemory (Munakata et al., 2012; Schneider & Löffler, 2016). Third, this improvement could be the result of the strengthening of top-down processing pathways due to experience, maturation, or both. Finally, these developmental changes could be attributed to children’s growing literacy and the hypothesized effects that this might have on lexical prediction (Mani & Huettig, 2014). While the current study cannot tease apart these possibilities, they are distinct predictions that could be empirically tested.

In the remainder of this Discussion, we will do three things: First, we return to a previous reading study that used a speeded cloze task with children and discuss the relationship between our findings and theirs (Lee et al., 2023). Second, we reconcile our findings with prior work suggesting that children demonstrate poor lexical prediction. Finally, we briefly discuss limitations of the current study, including how our results relate to research on children’s moment-to-moment language comprehension.

### 4.1. Comparing our study to a prior cloze task with children

Lee and colleagues (2023) recently adapted a speeded cloze task design to explore the mechanisms underlying children’s predictions. Adults and children (4–12 years, *Mean Age* = 9) read sentence fragments and then verbally produced a completion as quickly as possible. Some fragments strongly predicted a particular continuation (high constraint) and some did not (low constraint).

Both adults and children were faster to produce high cloze responses. Adults were also faster to respond in high constraint contexts, regardless of the cloze value of the word that they produced. In contrast, children were faster in high constraint contexts but only when the word they produced was high cloze. Children’s low cloze responses were slow regardless of the context’s degree of constraint. The authors present a few explanations for this pattern, including that children might simply consider fewer candidates for each sentence continuation. This explanation was tentatively supported by children in their study providing fewer unique responses to each item than adults did. Interestingly, we find the opposite pattern: Relative to adults, children provided more unique responses to both high cloze items (children = 6, adults = 3) and low cloze items (children = 10, adults = 6). The different response patterns in these two studies could reflect various things: How children respond in the presence versus absence of time pressure, the richness of extended discourses versus sentential contexts, reading versus auditory comprehension, or the use of naturally paced materials versus word-by-word presentation.

The current study differs from Lee et al. in several notable ways^2^, and addresses a different question. Their results do, however, support our conclusion that children can make lexical predictions in a cloze task.

### 4.2. Reconciling our findings with the predictive processing literature

The present study demonstrates that children can make robust predictions about which word might come next in a predictable context. As we noted in the Introduction, most studies on prediction in children use measures that cannot tell us *what* is being predicted (event properties, upcoming referents, semantic features of upcoming words, or the words themselves). There are, however, two lines of work that have targeted prediction at the lexical level. The first demonstrates that gender-marked determiners can facilitate the recognition of nouns in toddlers, children, and adults in a two-alternative preferential looking task (Lew-Williams, 2017; Lew-Williams & Fernald, 2007). But because these effects emerge after noun onset (even in adults), it is unclear whether they reflect rapid prediction of the noun or integration of gender cues after encountering the noun. The present findings are compatible with either interpretation. If we take these results as evidence of prediction, then the present study goes beyond those findings by showing that children can predict words even when doing so requires more than selecting between two possible objects.

The second line of work, by Gambi and colleagues (2018), provides a more direct contrast with the present study. As noted earlier, this study used a two-alternative preferential looking task in which the critical determiner in the test sentences could be used to predict the form of the upcoming noun, potentially resulting in anticipatory looks to the intended referent. Critically, in contrast with the studies above, the researchers inserted a pause between the determiner and the noun, resulting in a 1200 ms time window during which predictive looks could be measured. While adults anticipatorily shifted their gaze to the intended referent, 2- and 5-year-old children did not, suggesting that they failed to predict based on the determiner.

Given these findings, our results are somewhat surprising. At first blush, our task seems like a more challenging measure of prediction. While Gambi et al. (2018) narrowed down the world of possible continuations to just two words, there were thousands of potential continuations in our task (in the absence of predictive constraints). In their task, participants only needed to attend to one information source (the determiner) to predict the target. In contrast, our participants needed to integrate a variety of constraints from a rich, complex, and gradually unfolding discourse. Finally, while Gambi et al. (2018) used eye movements to measure implicit prediction, our study required children to make explicit predictions.

We considered several explanations for these different findings. One possibility is that children can predict if given infinite time to do so but not in an online task. We find this unlikely. Because of the pause inserted in the sentences, children in Gambi et al. (2018) had 1200 ms to launch a predictive saccade before the beginning of the target word. This timing seems more than sufficient for generating predictions – in our study (after a few trials of exposure), children often began speaking as soon as the cartoon stopped. The second possibility is that children can make predictions at the lexical level but do so only when they are explicitly required to. We cannot rule out this possibility. However, it is worth noting that, on this hypothesis, lexical prediction would be radically different than referential prediction, which seems to occur spontaneously across a wide range of contexts. This hypothesis would be consistent with theories in which lexical prediction is more fragile and task dependent than semantic prediction (e.g., Ito, 2024; Nieuwland et al., 2018). In adults, implicit form-based prediction appears to be present in everyday discourse contexts (Yacovone et al., 2025). Parallel studies with children could clarify whether this is also true early in development.

The third possibility is that the difference in findings is related to the validity of the predictive cues in each task. Lexical prediction in Gambi et al. (2018) hinges on the distinction between *a* and *an.* In everyday contexts, this cue will not be particularly useful for two reasons: First, in ordinary speech, an indefinite determiner occurs almost immediately before the word that it constrains with no pause. Thus, any prediction solely based on the determiner would have to happen in an instant. Second, this determiner provides minimal information. Indefinite determiners like *a* and *an* divide the hypothesis space for upcoming words into two sets: Words that begin with a vowel and words that do not. Each of these sets contains thousands of potential lexical candidates. In contrast, lexical prediction in the present study relied on a rich set of discourse cues. Although these story-based cues had greater complexity, they were highly constraining, often pointing participants toward a single upcoming word. The contextual constraints in our story were often present as early as four words before the target, allowing predictions to build up over roughly 1–2 seconds (Yacovone et al., 2025). Thus, children may come to use the discourse context as a predictive cue because it is highly informative, easily accessible, and available early.

### 4.3. Addressing limitations of the current study

There are two primary limitations to the present study. First, while our findings demonstrate that children can make explicit predictions about which word is likely to occur next they do not demonstrate that children predict quickly enough to facilitate comprehension in real time. Prediction is inherently a top-down process, and successful prediction requires constructing and building high-level representations of the unfolding context in order to predict upcoming linguistic representations. Thus, to explore the utility of children’s predictive processing, future research will need to focus on whether children rapidly predict during online comprehension. Recent work using electroencephalography (EEG) finds that the amplitude of the N400 in 5- to 10-year-old children is correlated with cloze probability during passive story listening (Levari & Snedeker, 2024). This suggests that children within this age range are predicting during online comprehension. Future work should finely explore this age range to determine when online prediction emerges – the current study finds that offline lexical prediction is present by age 5. Future work should also investigate children’s predictions at different levels of linguistic representation across development.

Second, participants in the current study viewed a cartoon while listening to the story. Consequently, we cannot conclude that prediction would be as accurate in a context where only linguistic input was available. To be clear, we do not think that participants in the current study were simply selecting their response from the set of objects that happened to be on the screen before the cartoon paused. If they were, then we would expect performance to be extremely poor for the trials on which the referent of the noun was not on the screen immediately prior to guessing. Instead, we found that children’s performance on these trials was quite high in the high cloze condition (see Figure 6). Thus, the visual context in the present study serves a very different function from the context in a visual world paradigm study. In these studies, the set of pictures constitutes the total set of referents or response options. In our study, the cartoon illustrated ongoing actions and thus provided additional support for constructing an accurate representation of the discourse. This discourse representation constrained lexical prediction. But the scene itself did not strongly constrain responses. We might, therefore, expect that young children would succeed at prediction, even in the absence of a visual context, provided that they were able to rapidly and accurately represent the evolving discourse. This hypothesis, however, remains to be tested.

## 5. Conclusion

Adults can predict specific upcoming lexical items before they appear in the input during language comprehension. While previous studies find that children have expectations about upcoming content, prior work was ambiguous with respect to the nature of the representations being predicted. The current study contributes to this literature by using a spoken, story-based version of a classic cloze task to demonstrate lexical prediction in children. We find that children (5;0–6;11) make robust lexical predictions, correctly providing the exact target word, out of all the words in their lexicons, over half the time in predictable contexts. Furthermore, when children fail to produce the exact target, they demonstrate a sensitivity to the context by often providing words that are semantically related to the target. We also found tentative evidence that prediction improves between five and six. These findings motivate studies exploring prediction across a wider age range to understand the developmental trajectory and research using passive listening tasks to understand children’s prediction during online comprehension.

## Data Availability

Pre-registrations, data, and code are publicly available on Open Science Framework (OSF https://osf.io/dgbv3/).

## Footnotes

1 Gambi and colleagues (2018) report statistically reliable effects of condition in their 3- to 4-year-olds and 5-year-olds but no Time by Condition interaction. The effects emerge before the determiner and are quite small. We believe that these are false positives that can be attributed to the anti-conservativity of growth curve models as applied to eye-tracking data (see Huang & Snedeker, 2020).

2 Our study did not require reading, allowing us both to explore predictive processing in spoken language and focus on a younger age group. We used a complete story discourse, rather than individual sentence fragments. Finally, the authors do not report overall accuracy, which would be the most direct comparison to the current study.
